# Watching individual molecules flex within lipid membranes using SERS

**DOI:** 10.1038/srep05940

**Published:** 2014-08-12

**Authors:** Richard W. Taylor, Felix Benz, Daniel O. Sigle, Richard W. Bowman, Peng Bao, Johannes S. Roth, George R. Heath, Stephen D. Evans, Jeremy J. Baumberg

**Affiliations:** 1 NanoPhotonics Centre, Cavendish Laboratory, Department of Physics, JJ Thompson Ave, University of Cambridge, Cambridge, CB3 0HE, UK; 2 Department of Physics, University of Leeds, Leeds, UK; 3 These authors contributed equally to this work.

## Abstract

Interrogating individual molecules within bio-membranes is key to deepening our understanding of biological processes essential for life. Using Raman spectroscopy to map molecular vibrations is ideal to non-destructively ‘fingerprint’ biomolecules for dynamic information on their molecular structure, composition and conformation. Such tag-free tracking of molecules within lipid bio-membranes can directly connect structure and function. In this paper, stable co-assembly with gold nano-components in a ‘nanoparticle-on-mirror’ geometry strongly enhances the local optical field and reduces the volume probed to a few nm^3^, enabling repeated measurements for many tens of minutes on the same molecules. The intense gap plasmons are assembled around model bio-membranes providing molecular identification of the diffusing lipids. Our experiments clearly evidence measurement of individual lipids flexing through telltale rapid correlated vibrational shifts and intensity fluctuations in the Raman spectrum. These track molecules that undergo bending and conformational changes within the probe volume, through their interactions with the environment. This technique allows for *in situ* high-speed single-molecule investigations of the molecules embedded within lipid bio-membranes*.* It thus offers a new way to investigate the hidden dynamics of cell membranes important to a myriad of life processes.

Lipid membranes that constitute the surface of cells perform vital biological functions, from molecular trafficking to signaling^1^,^2^. Composed predominantly of organic lipids, these bio-membranes have become an intense area of research across many disciplines as efforts are made to understand the complex physiochemical dynamics of the lipids^3^,^4^. Moreover, the cell membrane is built up of a complex mixture of phospho- and glyco-lipids as well as numerous proteins that interact with each other in sophisticated cascades that sustain biological pathways. To probe and identify non-destructively at the single molecule level the interactions on these membranes would be an invaluable investigative and diagnostic tool. Not only would it enable greater understanding of the interaction of drugs and viruses that incorporate within the cell membrane, but also important fundamental life processes such as membrane budding and fusion. Furthermore many degenerative diseases, such as Alzheimer's, Parkinson's, cystic fibrosis and muscular dystrophy are believed to result from damage to the cell membrane. Probing single molecule behaviour in lipid bilayers can thus track in depth the development of these diseases.

To observe *in situ* the dynamic changes in conformation and orientation of single lipids composing the bio-membrane is extremely challenging. Raman vibrational spectroscopy is ideally suited to provide information sensitive to the molecular species, orientation of constituent dipole moments, chemical state and local stresses in the form of a unique vibrational ‘fingerprint’^5^,^6^. The challenge for single-molecule spectroscopy of biological systems is to provide a localized enhancement of the Raman signal sufficient to allow measurement with high-enough signal to noise, and to do so on the sub-second time scales necessary for following membrane dynamics. It is also important that this probe does not damage the delicate biological sample, allowing continued observations.

Placing gold nanoparticles extremely close to a gold mirror (Fig. 1a) forms a construct well-suited to tightly confine light within a gap of nanoscale dimensions, in a ‘gap plasmon’. Such gap plasmons have been used for sensitive spectroscopy of graphene monolayers^7^ and gap-localized chemical reactions^8^ with concentrations in the zeptomolar range^9^,^10^,^11^. This gold nanoparticle-on-mirror (NPoM) system is ideally suited for enhanced vibrational spectroscopy due to the biological compatibility (inertness) of gold and the ability to create field volumes only a few molecules across^3^,^4^,^12^. Its advantage over previous work utilising metal tip-enhanced Raman (TERS) is not only its greater stability but also its ease of use while having a similar or even higher spatial resolution and field enhancement^12^,^13^,^14^.

In this work, as a model for the biomembrane we use a mixture of the lipids 1-palmitoyl-2-oleoyl-sn-glycero-3-phosphocholine (POPC) and 1,2-dioleoyl-3-trimethylammonium-propane (DOTAP), in an 80:20 concentration ratio. Phospholipids with the choline group are chosen as our model because they are one of the most abundant lipid species in eukaryotic cells^15^. This lipid leaflet is supported on a self-assembled monolayer (SAM) of long tail alkanethiols (octadecanethiol, ODT) to form a hybrid bilayer leaflet (HBL, Fig. 1a), a model analogue to the cell membrane^16^. The HBL is kept hydrated in buffer solution to preserve the uniform membrane structure. At room and biological temperatures the POPC:DOTAP leaflet is in the fluid phase. The low steric forces of this phase allow for a lower mean tail density, greater tail disorder and high mobility of the lipid^17^. This easier lipid movement is then investigated via the gap-plasmon-enhanced Raman signature.

The gap plasmon cavity is formed between a flat Au surface and 80 nm diameter gold nanoparticles deposited *in situ* on top of the supported HBL. Illuminating gold particles with a high numerical aperture objective excites the dipole mode orthogonal to the surface, and this dipole strongly interacts with its image in the planar gold surface. The resonance wavelength of this hybridized gap mode is exquisitely sensitive to the separation of the particle from the plane surface. Viewed under dark-field scattering microscopy (Fig. 1b,c, Fig.S1), the gap mode wavelength of the NPoM HBL is observed at a well-defined wavelength 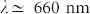, permitting efficient Raman laser excitation with λ_R_ = 633 nm. This gap mode wavelength matches our calculations for plasmonic coupling across a gap of 5 nm, corresponding to the height of the HBL of 4.4 ± 0.6 nm measured by atomic force microscopy (AFM, Fig.S3), with a dielectric constant *ε_r_* ~ 2^18^. The reproducibility of the gap mode is attributed to the ordering of the HBL spacer. The variations in particle colour seen in Figure 2(b) are due to differences in height across the HBL, revealed by AFM (see Supp. Info.). The smoothness of the gold surface is measured to be <0.5 nm. Each nanoparticle initially diffuses around before settling on the lipid surface and becoming red under scattering microscopy (Fig. 1c, 1–3, Fig.S1). Some nanoparticles however remain further spaced (green, Fig 1c, 4). We perform all spectroscopy on particles observed to be stationary and with the 660 nm gap plasmon resonance.

The stability and reproducibility of the gap plasmon field demonstrated above enables reproducible measurement of the POPC:DOTAP membrane, shown in averages of repeated time series (Fig. 2a). The field enhancement of the gap generates sharp vibrational modes with high signal-to-noise, which can be assigned to the known tail alkane vibrations and also the choline head group for the membrane lipids. A distinct feature of HBL spectroscopy from the NPoM gap plasmon is the high density of vibrational modes observed, attributed to the tight confinement of the cavity field around the probe molecules. Similar traits are also observed in single molecule SERS using gap plasmons between two nanoparticles where the field is similarly confined^19^,^20^,^21^. Here the field is further confined within the HBL due its dielectric contrast with the surrounding water. For plasmon fields with lower spatial confinement, such as with TERS or plasmonic substrates such as Klarite^TM^, this spectral density is not observed (Fig.S4 and [22–25]). The rich vibrational spectrum from the gap plasmon construct is due to escaping the typical averaging over molecular dynamics in SERS, as discussed below. It is also partly attributed to modified Raman selection rules induced by the high degree of alignment of lipids normal to the planar Au surface along the very strong gradient in optical field^22^,^26^. This abundance of vibrational modes, each sensitive to the highly-polarized gap field orientated perpendicular to the planar surface, clearly enables the SERS spectroscopy to report minute changes in the orientation, conformation and composition of the probed molecules^27^.

The discrimination in lipid species afforded by the NPoM gap plasmon is demonstrated by comparing hydrogenated DOTAP (20%) and deuterated POPC (80%) in the HBL (Fig. 2b). These time-ensemble vibrational spectra reveal a series of bands in the D-POPC HBL spectrum (highlighted) downshifted in frequency from those of H-POPC. The Raman bands at 3000 cm^−1^ and 900 cm^−1^ originate respectively from **CH_2_**/**CH_3_** stretches and **CH_2_** twisting/wagging of the alkyl tails^28^,^29^. In the deuterated analogue, the hydrocarbon **CD** groups have a greater mass and thus a lower vibrational frequency that is clearly observed in the enhanced Raman spectrum. In addition, the D-POPC lipid species possess some vibrational modes unaffected by the deuterium substitution. Accordingly these vibrations occur at the same frequency in both spectra (**C–C** stretch 1010 cm^−1^ and **N-H** stretch 720 cm^−1^), confirming the discriminatory ability of the NPoM SERS^30^.

The high field enhancement afforded by the gap mode allows for fast acquisition (<1s) of vibrational spectra so that time-dependent dynamics of the fluid bilayer may now be tracked. As an example of this (Fig. 2c) we measure the gap plasmon SERS from an HBL containing also a third lipid in low concentration (PTPC, 5%). This PTPC lipid contains a triple carbon bond in one tail with a distinct unambiguous Raman vibration at 2200 cm^−1^. By continuously measuring the SERS spectrum from a single NPoM over time, the diffusion dynamics of PTPC within the membrane is observed. The acetylene signature vibration of PTPC fleetingly appears as the lipid molecules diffuse into and out of the gap plasmon mode volume intersecting the membrane. The evolution of this signature mode (Fig. 2c inset) over 300 s (normalized to the 1380 cm^−1^ **CH_2_** reference) implies that single molecules can be seen dynamically as we discuss below.

The sensitivity of the measured SERS spectrum to the probed molecular conformation and orientation within the field allows for complex and varied molecular flexing and movement to be observed. We are able to reliably record SERS trajectories from each individual gold NPoM spanning the lipid membrane, for times of order many hours, with SERS intensities remaining strong (Fig.S6). In Figure 3a, a section of such a time series is seen which shows that far from being static over the course of measurement, specific Raman vibrations of the lipids undergo fluctuations in intensity and spectral position. Analysis of these data sets reveal some clear patterns, with two features implying that only few molecules are being probed: (a) opposite simultaneous shifts in vibrational frequency of different lines can be seen, and (b) a number of lines can appear and disappear simultaneously.

The Raman intensity for each particular line varies, but in a manner uncorrelated to other Raman lines. Such observations are consistent with single-molecule measurements^31^,^32^ and, although a ‘blinking’ mechanism is not yet clear, it may arise from perturbations of the molecular excited state lifetime^31^ or the Raman vibronic coupling^24^. More interesting are the simultaneous correlated shifts in Stokes frequencies of different Raman modes, seen for the first time. The variation between different vibrational lines in the frequency shifts and directions strongly implies that vibrations seen here are localized to particular parts of each molecule.

A summary of our measured *rms* mode shifts on the narrowest lines averaged over 1000 spectra (Table 1) reveals shifts of magnitude 2–6 cm^−1^. Compression from metal tips in TERS has been suggested to shift modes, with estimates that in adenine molecules 10% bond contraction yields a 5 cm^−1^ shift^15^,^25^,^33^. This flexing of the lipid molecules within the probed gap thus causes both amplitude and frequency fluctuations. Conventional measurements average over all such dynamical and local information, but single-molecule SERS using gap-plasmons can reveal these synchronous shifts, further discussed below. The ever-present 1380 cm^−1^ **CH_2_** deformation seen is common to all membrane molecules and since these modes are very localized in the chains it is least sensitive to larger structural deformations – we thus use it as normalization for the total number of molecules probed.

We discuss now typical key events (Fig. 3) from many among the full data sets (for others see Fig.S7). One dramatic event (typically every few minutes) is a sudden change in the entire Raman fingerprint (Fig. 3a). Assigning the molecular bonds for each peak allows this event to be understood as a change in conformation of the lipid molecule. Beforehand, clear Raman signatures of the lipid tail, ν(**C–C trans**) at 1139 cm^−1^ and ν(**C = C**) around 1550 cm^−1^, are seen as well as the characteristic mode of the ester linking group ν(**-[C = O]-O-C**) at 1183 cm^−1^.

Initially the small frequency shifts in these lines are correlated (Fig. 3a, right), implying they originate from a single lipid molecule. During the rapid (<1s) dramatic perturbation of the SERS spectra, bands disappear while new bands emerge. These include the characteristic mode of the phosphate group ν(**PO_2_**) around 800 cm^−1^ and a clearly shifted trans ν(**C–C**) at 1120 cm^−1^. The stretch of the double bond ν(**C = C**) centred around 1550 cm^−1^ persists, suggesting a change in the orientation of the lipid head group with respect to the optical field polarization in the gap. This change in conformation can be followed only because of the precisely defined field orientation in our gaps and the well-defined Raman dipole moments of the lipids. Chemical transformations may also induce such reconfigurations, but are not considered likely in the environment here. Along with the spectral rearrangement, a strong persistent increase in SERS amplitude is also seen which may arise from the improved alignment of Raman dipoles with the gap field.

More intricate changes in lipid conformation can be observed on the timescale of seconds (Fig. 3b). We refer to correlated changes in vibrational modes as ‘molecular flexing’, and explore the changes within events **I** and **II**. Within **I**, three bands are seen that exhibit synchronous frequency shifts with a similar magnitude of 10 cm^−1^ (from the previous 1183, 1550 bands and also one at 1070 cm^−1^), suggesting they all report different aspects of a global molecular deformation. The line at 1070 cm^−1^ reports a *gauche* to *trans* conformational change of the alkane tail (**C–C**)^27^. Such discrete switching is possible only if a single molecule is being interrogated, unless several lipids move completely in tandem which is very unlikely given the fluidity and activity of the membrane. Changes in leg conformation increase the strain of this lipid inside the fluid leaflet and this strain is reflected in the downshift of the 1183 cm^−1^ ester linking group. However while this molecular flex takes place, some modes of the lipid remain unaffected, such as the 1380 cm^−1^ **CH_2_** deformation of the alkane legs. On the other hand, correlated shifts for choline, ester, and alkene bonds suggest strain between the head group (which is strongly attracted to the nanoparticle) and the tail which is ‘jittering’ in the membrane beneath.

The SERS trajectory then shows a rapid evolution to fingerprint **II** (Fig. 3b) which is coupled to the events within **I**, and interpreted as arising from the same molecule. Enhancement of the 1325 cm^−1^ methyl deformation (lipid head group) mode likely reflects its realignment to the optical field direction, but its simultaneous spectral shifts are anti-correlated with the carbonyl group stretch [ν(**C = O**), around 1700 cm^−1^]. We find in **II** clear anti-correlations (with spectral line shifts mirrored in opposite directions) in the head deformation band at 1320 cm^−1^ and carbonyl stretch at 1700 cm^−1^ (Fig. 3d)^34^. These spectral dynamics, which can only be explained as arising from individual lipids, highlight our capability to now measure molecular flexing in real time, and indicate the large number of ways strain can be partitioned between parts of a molecule. Our ability to collect long time sequences of molecular flexing requires advances in theoretical tools to directly extract conformational dynamics. However the features highlighted here already clearly signal the wealth of information on the membrane that can be adduced. We analyse correlations in the spectral shifts between pairs of vibrations (*ν*_1_, *ν*_2_), restricted within shorter (30s) time spans on a single molecule, showing in Fig. 3(d,i) both positive (orange) and negative (purple) dependencies. We extract all of these correlations given by slopes *g_ab_* = Δ*ν_b_*/*ν_a_*, and capture in Fig. 3(d,ii) this ‘correlation fingerprint’ as *c_ab_* = sin(2 atan *g_ab_*) which records how close the two frequency shifts are perfectly locked (+1) or inverted (-1) or uncorrelated (0). The size of each *c_ab_* point records the correlation coefficient of the linear regression (how good their correlation is). In general we find positive correlations are stronger than negative ones, reflecting the local response of neighbouring bonds. This can be quantitatively addressed by plotting the correlation strength for the assigned bonds *vs* their separation, in terms of number of atoms, in Fig. 3(d,iii). Typically we find decreasing correlation strength at more remote separations. These correlations extracted from the flexing dynamics thus encode detailed information on how molecules move.

Besides key questions about the rate of molecular flexing (previously hidden) for which we are developing faster spectroscopies, and how it varies in different lipid layers, fundamental puzzles remain about the success of the technique. Brownian motion would be expected to move molecules through the probe volume within 10 μs, hence pinning of the charged DOTAP rafts^25^ and interactions with the Au nanoparticle must retard lipid motion. Such pinning is consistent with the observation of reduced diffusion of membrane lipids bearing adsorbed charged polymers on the membrane surface^35^. In addition, similar to recent TERS^12^,^23^,^31^, the lateral resolution observed exceeds predictions based on simple models of the optical field in nanogaps between smooth surfaces and sharply bounded metals (Fig.S8). The influence of atomically-sharp corners on the facetted nanoparticles may play a role in further field localization, leading to single nanoparticle SERS over a few lipid molecules. Such fields can also exert optical forces at the nanoscale which influence molecular diffusion.

Vibrational spectroscopy of single molecules within lipid bio-membranes presents a powerful means to explore and characterize molecular interactions at the nanoscale. Real-time spectroscopies will enable greater understanding of the precise interaction of proteins and lipids in the dynamic and complex cell membrane surfaces. Our findings here open the possibility for monitoring of other cell processes relevant to fundamental cellular operation at the single molecule level in biological environments.

## Methods

### Sample Preparation

NPoM - HBL formation: A flow cell chamber was fabricated from PDMS with a thin 10 μm glass coverslip to provide a viewing window. Gold of thickness 50 nm was evaporated onto 10 nm Ti/350 nm SiO_2_/500 μm silicon by e-beam evaporation. The rms roughness was determined to be less than 0.5 nm. The ODT monolayer was formed by submerging the substrates in a 5 mM ODT solution in dichloromethane (DCM) for 24 h at room temperature. The samples were thoroughly rinsed with DCM and dried under nitrogen flow. The contact angles of deionized water on this surface (advancing: 110.0° and receding: 104.2°) were obtained by a FTA4000 Microdrop goniometer (First Ten Angstroms, Inc). The thickness of the ODT SAM (2.1 ± 0.1 nm) on Au was characterized by a UVISEL Spectroscopic Ellipsometer (Horiba, Ltd). The rinsed ODT-Au SAM was placed within a home-built flow cell and lipid solution flowed into it and left for 1 hour. The excess was rinsed with a tris(hydroxymethyl)aminomethane (TRIS) buffer solution and the AuNPs (80 nm diameter, BBI Solutions, UK) similarly flowed through, rinsed and removed after 1 hour. Within several minutes, accumulation of AuNPs on the surface was observed, and the times were adjusted to ensure a suitable surface density for the experiments.

### Experimental

Single particle scattering spectroscopy: White light scattering spectroscopy was performed *in situ* on the membrane with fibre-coupled illumination from a 40 W halogen lamp (≈60° incidence angle). The scattered light was collected with a long working distance x50 objective (of the Raman microscope) and fibre-coupled into a TEC-cooled OceanOptics QE65000 spectrometer. The integration time was 1 s.

Raman spectroscopy: A Renishaw InVia Raman microscope was used for all measurements in point scan mode on the flow cell sample. A x50 long working objective was used to collect all spectra. A grating of 1200 lines/mm was used with an appropriate edge filter. Excitation of Raman modes was with 633 and 785 nm HeNe and diode lasers, typically ≈1 mW.

## Author Contributions

Experiments were planned and executed by S.D.E., R.W.T., F.B., D.O.S., R.W.B., G.R.H. and J.J.B., with support for lipid assembly from P.B. and J.R. The data were analysed by F.B., R.W.T., P.B., J.R., S.D.E. and J.J.B. and all authors contributed to the manuscript.

## Supplementary Material

Supplementary Information Supplementary Information

## Figures and Tables

**Figure 1:**
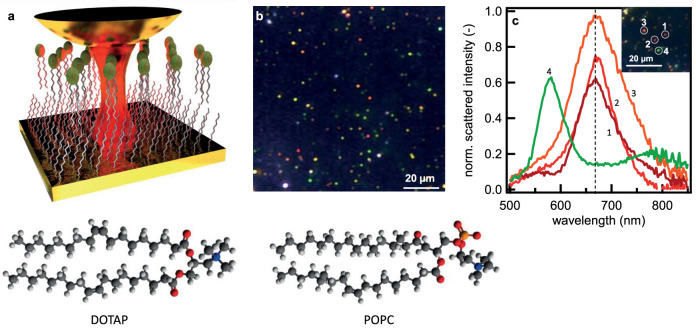
Formation and characterization of gap plasmon sensor. (a), Schematic of gold nanoparticle (diameter *d* = 80 nm) deposited upon lipid-alkanethiol hybrid bilayer on a planar gold surface, in solution. Lipids are POPC:DOTAP and SAM is octadecanethiol. (b), Wide-field scattering image of gold nanoparticles on lipid hybrid layer. (c), Reproducible scattering spectra of the gap plasmon resonance from individual gold particles (shown in inset) on the hybrid lipid layer.

**Figure 2:**
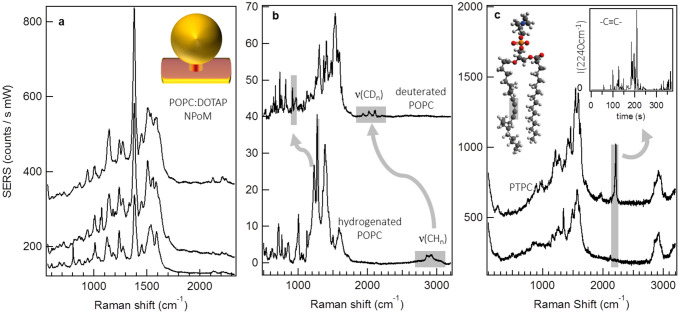
Lipidomics of lipid layers with gap plasmon SERS. (a), SERS spectra (1 s acquisition time, averaged over 300 subsequent spectra) for three separate particles on a POPC:DOTAP 80:20 hybrid bilayer. (b), SERS spectrum of D-POPC:H-DOTAP (10 s acquisition time) highlighting Raman modes shifted due to deuteration. (c), Selected spectra from repeated measurement of a three component lipid layer including 5% PTPC possessing unique acetylene signature mode highlighted in molecule. Inset: SERS intensity of PTPC acetylene mode with time (normalized to CH_2_ stretch intensity and acquisition time 1 s). All spectra in a–c acquired with 1 mW excitation at λ_R_ = 633 nm.

**Figure 3:**
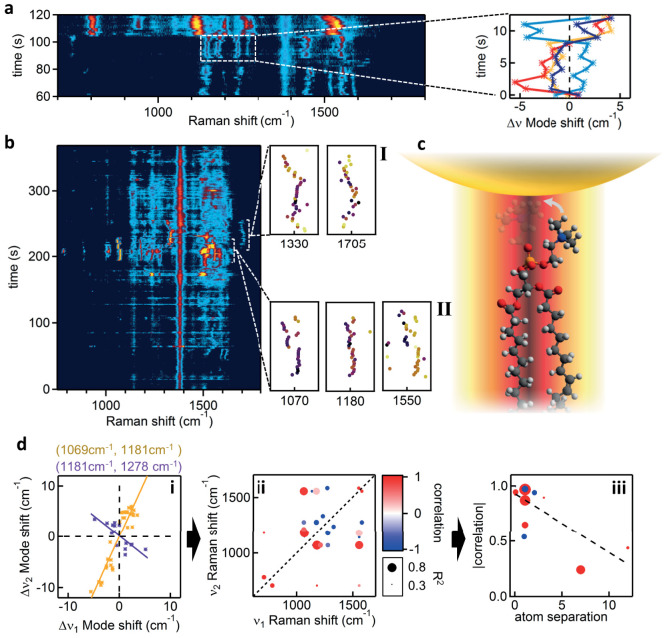
Fast dynamics of hybrid lipid-bilayer (POPC:DOTAP) SERS. (a), Time dependent evolution of SERS from a single gold NPoM. Trajectories of individual lines show dramatic jumps and correlated shifts, corresponding to lipid dynamics within the gap plasmon volume. Right: Selected short-time dynamics highlighting the (anti-)/correlations between Raman lines. (b), Raman trajectory, including flexing events labelled **I**, **II** extracted to right. (c), Schematic lipid flexing within gap plasmon hot spot. (d), **i,** Frequency shifts at each time step of Raman line pairs (*ν*_1_, *ν*_2_) showing positive (orange) and negative (purple) correlations. **ii,** Strength of correlation between pairs of Raman lines (*ν*_1_, *ν*_2_) with size of points giving correlation strength R^2^. **iii,** Magnitude of correlation (as in ii) when separation between bonds involved increases (dashed linear fit has R^2^>0.5). All spectra acquired at λ_R_ = 633 nm with acquisition time of 1 s.

**Table 1:** Average flexing mode-shifts for selected assigned Raman modes. The central mode frequency and the *rms* magnitude of temporal frequency shifts is measured from a sample of 1000 measurements (ν = stretch, δ = bend)

Mode assignment	Centre Frequency (cm^−1^)	*rms* mode-shift (cm^−1^)
δ(**CH_2_**)	1339	6
ν(**-C = O**)	1700	6
ν(**–[C = O]-O-C–**)	1183	2.3
ν(**PO_2_**)	1239	3.0

## References

[b1] Van Meer G., Voelker D. R. & Feigenson G. W. Membrane lipids: where they are and how they behave. Nat. Rev. Mol. Cell Biol. 9, 112–124 (2008). 18216768 10.1038/nrm2330 PMC2642958

[b2] Sprong H., Sluijs P. Der & Van G. How proteins move lipids and lipids move proteins. Nat Rev Mol Cell Biol. 2, 504–513 (2001). 11433364 10.1038/35080071

[b3] Dietrich C. *et al.* Lipid rafts reconstituted in model membranes. Biophys. J. 80, 1417–1428 (2001). 11222302 10.1016/S0006-3495(01)76114-0 PMC1301333

[b4] Baumgart T., Hess S. T. & Webb W. W. Imaging coexisting fluid domains in biomembrane models coupling curvature and line tension. Nature 425, 821–824 (2003). 14574408 10.1038/nature02013

[b5] Edidin M. Lipids on the frontier: a century of cell-membrane bilayers. Nat. Rev. Mol. Cell Biol. 4, 414–418 (2003). 12728275 10.1038/nrm1102

[b6] Jacobson K., Mouritsen O. G. & Anderson R. G. W. Lipid rafts: at a crossroad between cell biology and physics. Nat. Cell Biol. 9, 7–14 (2007). 17199125 10.1038/ncb0107-7

[b7] Mertens J. *et al.* Controlling subnanometer gaps in plasmonic dimers using graphene. Nano Lett. 13, 5033–5038 (2013). 24059599 10.1021/nl4018463

[b8] Taylor R. W. *et al.* In situ SERS monitoring of photochemistry within a nanojunction reactor. Nano Lett. 13, 5985–5990 (2013). 24188432 10.1021/nl403164c PMC3883114

[b9] Lingwood D. & Simons K. Lipid rafts as a membrane-organizing principle. Science 327, 46–50 (2010). 20044567 10.1126/science.1174621

[b10] Nie S. Probing Single Molecules and Single Nanoparticles by Surface-Enhanced Raman Scattering. Science 275, 1102–1106 (1997). 9027306 10.1126/science.275.5303.1102

[b11] Kneipp K. *et al.* Single Molecule Detection Using Surface-Enhanced Raman Scattering (SERS). Phys. Rev. Lett. 78, 1667–1670 (1997).

[b12] Zhang R. *et al.* Chemical mapping of a single molecule by plasmon-enhanced Raman scattering. Nature 498, 82–86 (2013). 23739426 10.1038/nature12151

[b13] Richter M., Hedegaard M., Deckert-Gaudig T., Lampen P. & Deckert V. Laterally Resolved and Direct Spectroscopic Evidence of Nanometer-Sized Lipid and Protein Domains on a Single Cell. Small 7, 209–214 (2011). 21213383 10.1002/smll.201001503

[b14] Yang Z., Aizpurua J. & Xu H. Electromagnetic field enhancement in TERS configurations. J. Raman Spectrosc. 40, 1343–1348 (2009).

[b15] Attwood S. J., Choi Y. & Leonenko Z. Preparation of DOPC and DPPC Supported Planar Lipid Bilayers for Atomic Force Microscopy and Atomic Force Spectroscopy. Int. J. Mol. Sci. 14, 3514–3539 (2013). 23389046 10.3390/ijms14023514 PMC3588056

[b16] Plant A. L. Supported Hybrid Bilayer Membranes as Rugged Cell Membrane Mimics. Langmuir 15, 5128–5135 (1999).

[b17] Leonenko Z. V., Finot, E., Ma, H., Dahms, T. E. S. & Cramb, D. T. Investigation of temperature-induced phase transitions in DOPC and DPPC phospholipid bilayers using temperature-controlled scanning force microscopy. Biophys. J. 86, 3783–3793 (2004). 15189874 10.1529/biophysj.103.036681 PMC1304279

[b18] Howland M. C., Szmodis A. W., Sanii B. & Parikh A. N. Characterization of physical properties of supported phospholipid membranes using imaging ellipsometry at optical wavelengths. Biophys. J. 92, 1306–1317 (2007). 17142265 10.1529/biophysj.106.097071 PMC1783900

[b19] Savage K. J. *et al.* Revealing the quantum regime in tunnelling plasmonics. Nature 491, 574–577 (2012). 23135399 10.1038/nature11653

[b20] Xu H., Aizpurua J., Kall M. & Apell P. Electromagnetic contributions to single-molecule sensitivity in surface-enhanced raman scattering. Phys. Rev. E. 62, 4318–4324 (2000). 10.1103/physreve.62.4318 11088961

[b21] Romero I., Aizpurua J., Bryant G. W. & García De Abajo F. J. Plasmons in nearly touching metallic nanoparticles: singular response in the limit of touching dimers. Opt. Express 14, 9988–9999 (2006). 19529393 10.1364/oe.14.009988

[b22] Sonntag M. D., Chulhai D., Seideman T., Jensen L. & Van Duyne R. P. The origin of relative intensity fluctuations in single-molecule tip-enhanced Raman spectroscopy. J. Am. Chem. Soc. 135, 17187–17192 (2013). 24079659 10.1021/ja408758j

[b23] Nakata A., Nomoto T., Toyota T. & Fujinami M. Tip-enhanced Raman spectroscopy of lipid bilayers in water with an alumina- and silver-coated tungsten tip. Anal. Sci. 29, 865–869 (2013). 24025569 10.2116/analsci.29.865

[b24] Lombardi J. R., Birke R. L. & Haran G. Single Molecule SERS Spectral Blinking and Vibronic Coupling. J. Phys. Chem. C 115, 4540–4545 (2011).

[b25] Li L., Hutter T., Steiner U. & Mahajan S. Single molecule SERS and detection of biomolecules with a single gold nanoparticle on a mirror junction. Analyst 138, 4574–4578 (2013). 23748709 10.1039/c3an00447c

[b26] Watanabe H., Ishida Y., Hayazawa N., Inouye Y. & Kawata S. Tip-enhanced near-field Raman analysis of tip-pressurized adenine molecule. Phys. Rev. B 69, 155418 (2004).

[b27] Yellin N. & Levin I. W. Hydrocarbon chain trans-gauche isomerization in phospholipid bilayer gel assemblies. Biochemistry 1, 642–647 (2001). 10.1021/bi00623a014 836805

[b28] Bunow R. & Levin W. Raman spectra and vibrational assignments for deuterated membrane lipids 1,2-Dipalmitoyl phosphatidylcholine-d9 and -d62. Biochim Biophys Acta. 489, 191–206 (1977). 579158 10.1016/0005-2760(77)90138-2

[b29] Gaber B. P., Yager P. & Peticolas W. L. Deuterated phospholipids as nonperturbing components for Raman studies of biomembranes. Biophys. J. 22, 191–207 (1978). 580768 10.1016/S0006-3495(78)85484-8 PMC1473445

[b30] Socrates G. Infrared and Raman Characteristic Group Frequencies: Tables and Charts. (John Wiley & Sons Ltd, Chichester, 2004).

[b31] Van Schrojenstein Lantman E. M., Deckert-Gaudig T., Mank A. J. G., Deckert V. & Weckhuysen B. M. Catalytic processes monitored at the nanoscale with tip-enhanced Raman spectroscopy. Nat. Nanotechnol. 7, 583–586 (2012). 22902959 10.1038/nnano.2012.131

[b32] Rodríguez-Lorenzo L. *et al.* Zeptomol detection through controlled ultrasensitive surface-enhanced Raman scattering. J. Am. Chem. Soc. 131, 4616–4618 (2009). 19292448 10.1021/ja809418t

[b33] Watanabe H., Ishida Y., Hayazawa N., Inouye Y. & Kawata S. Tip-enhanced near-field Raman analysis of tip-pressurized adenine molecule. Phys.Rev.B 69, 155418 (2004).

[b34] Böhme R. *et al.* Biochemical imaging below the diffraction limit--probing cellular membrane related structures by tip-enhanced Raman spectroscopy (TERS). J. Biophotonics 3, 455–461 (2010). 20535731 10.1002/jbio.201000030

[b35] Zhang L. & Granick S. Slaved diffusion in phospholipid bilayers. Proc. Natl. Acad. Sci. U. S. A. 102, 9118–9121 (2005). 15967988 10.1073/pnas.0502723102 PMC1166613

