# Supplementary material for: Watching individual molecules flex within lipid membranes using SERS
Source: Sci Rep. 2014 Aug 12;4:5940. doi: 10.1038/srep05940 (PMC4129422; doi:10.1038/srep05940)
Supplement: Supplementary Information [file srep05940-s1.pdf]

Supplementary Information

Watching individual molecules flex within model lipid bio-membranes using SERS

Richard W. Taylor, Felix Benz, Daniel O. Sigle, Richard W. Bowman, Peng Bao, Johannes Roth, George R. Heath, Stephen Evans*, Jeremy J. Baumberg*

S1. Dark-field scattering images

To characterise the nanoparticle on mirror (NPoM) construct, scattering spectra reveal the long-wavelength spectral resonance which tunes with gap size and refractive index filling. Additional dark-field images of 80nm gold nanoparticles on a hybrid bilayer of POPC:DOTAP on an ODT SAM are shown in Figure S1(a-c). The different colours of the nanoparticles indicate different heights above the gold substrate and different particle geometries. Both red/orange and green spots belong to individual nanoparticles. Whereas red nanoparticles sit directly on top of the hybrid lipid bilayer, green nanoparticles remain further away apart from the HBL. Yellow spots indicate scattering in the infrared, which is false colour-coded to yellow by the CCD camera. The brighter near-white spots correspond to small clusters consisting of several nanoparticles. The scattering spectra of several red nanoparticles are shown Figure S1(d). All nanoparticles show a transverse (uncoupled) mode around 550nm and a gap (coupled) mode around 700nm. These mode polarisations are illustrated in Figure S1(e).

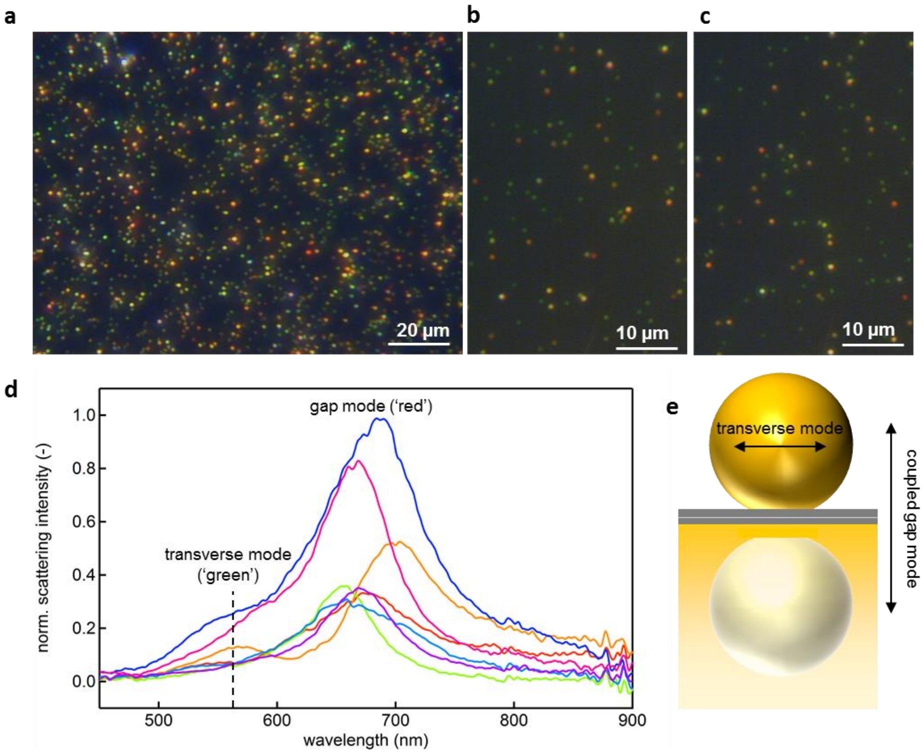

Figure S1 | Static characterisation of the gap plasmon sensor. a, Dark-field images recorded with a 50x and **b,c,** a 100x high numerical aperture brightfield objective. The sample was illuminated with a fibre-coupled 40 W halogen lamp from the side at an angle of approximately 60°. **d,** Scattering spectra of several red nanoparticles. The nanoparticles show both transverse (uncoupled) and longitudinal coupled gap modes, illustrated in **e**.

In order to investigate the stability of the nanoparticle assembly over time, dark-field spectra were continuously recorded with the same integration time as the SERS spectra. Figure S2(a) shows the acquired spectra over a time of 25s, together with the extracted line positions (Fig.S2b). The standard deviation of 0.5 nm is below the 6nm resolution limit of the spectrometer and unlikely to be significant.

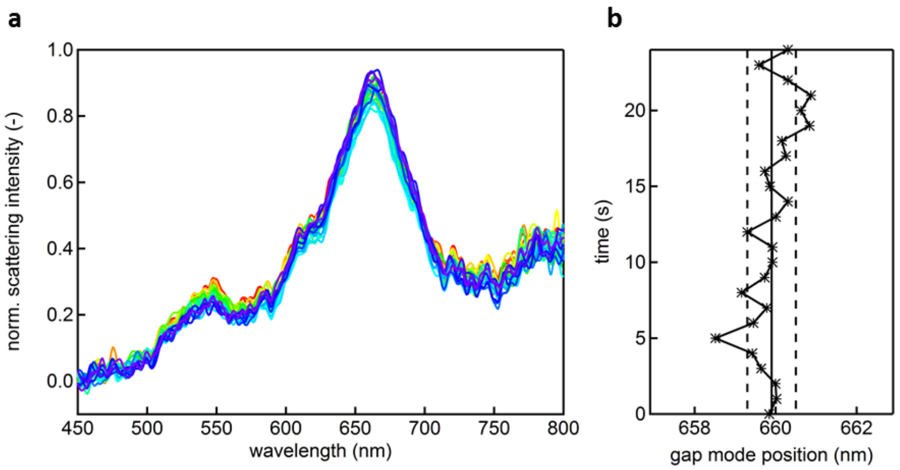

Figure S2 | Dynamic characterisation of the gap plasmon sensor. a, Time evolution of the scattering spectra from an individual gold NPoM with a POPC:DOTAP hybrid lipid bilayer, with 25 consecutive spectra recorded with 1s integration on the same nanoparticle. **b,** Extracted position of the gap mode over time. The solid line indicates the root mean square of the peak position, the dashed lines the confidence interval.

S2. Atomic Force Microscopy of lipid bilayers and Au NPs

Hybrid lipid bilayers (POPC:DOTAP 80:20 on alkanethiol SAMs) were characterised by AFM in order to obtain information on the morphology of the layer. Figure S3(a) shows an AFM image of an 80nm gold nanoparticle sitting on the hybrid bilayer. The line profile shown in Fig.S3(b) confirms the particle size. A relative large force is required to push a gold nanoparticle into the hybrid bilayer (Fig.S3c). The roughness of the hybrid bilayers is around 0.7 nm (Fig.S3d,e), while Fig.S3(f) shows the typical force profile expected for penetrating the hybrid bilayer with an AFM tip. These results confirm that the Au NPs sit on top of the lipid bilayer.

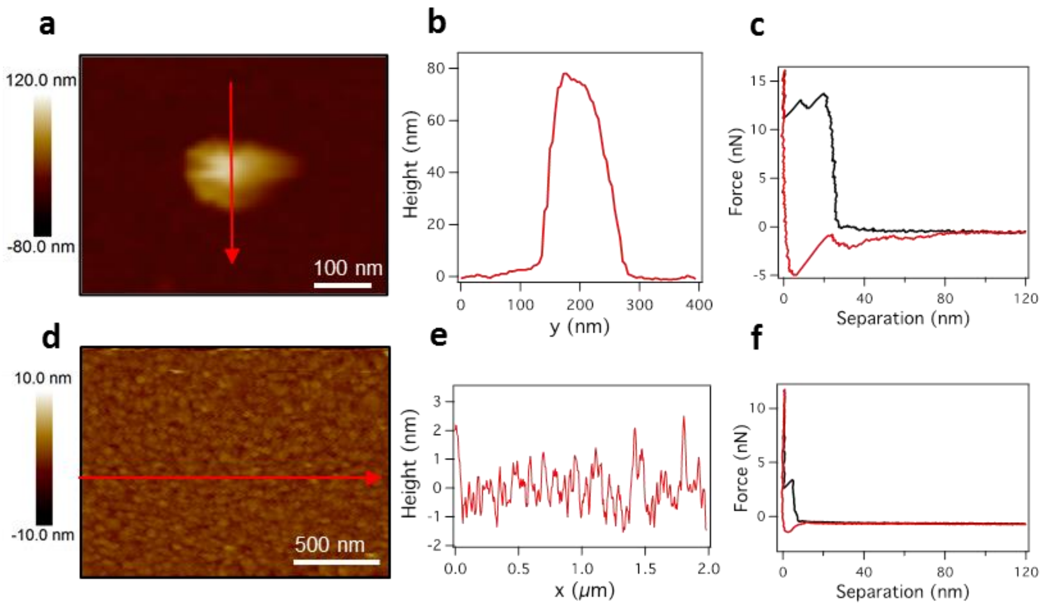

Figure S3 | AFM characterisation of hybrid lipid bilayers on gold. a AFM image of an 80nm gold nanoparticle on a hybrid lipid bilayer (POPC:DOTAP 80:20 on alkanethiol SAM on gold). **b,** Line profile across direction shown in **a**. **c,** Force profile on approach/retraction for the nanoparticle shown in **a**. **d,** AFM image of a hybrid lipid bilayer. **e,** Line profile across **d**. **e,** Force profile characteristic for a hybrid lipid bilayer.

S3. Phospholipid SERS measured from Klarite

Klarite™ is a commercial plasmonic substrate (from Renishaw Diagnostics) that enhances Raman scattering at the metal surface via plasmon resonance, but without the additional gap mode effects, and thus probes an very large number of molecules at the same time. The resulting SERS reference spectra of the phospholipids POPC and DOTAP, as well as a DDT alkanethiol SAM on Klarite, are shown in Figure S4. The line labelled K is found on the bare Klarite substrate and is a contaminant mode.

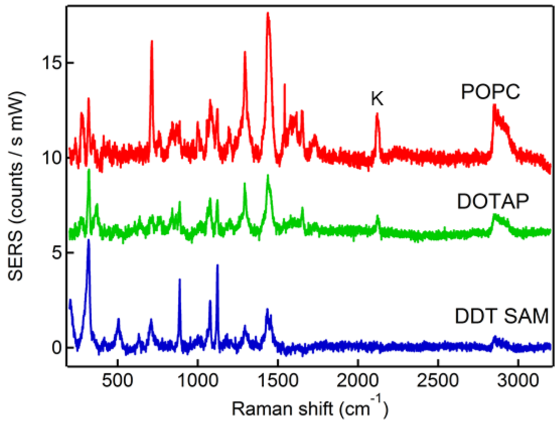

Figure S4 | Reference spectra on Klarite. SERS spectra of the two phospholipids POPC and DOTAP and the DDT alkanethiol SAM on Klarite. Laser 785nm, 100mW, 5s integration time.

S4. Plasmon Gap mode SERS of ODT

Reference measurements with ODT alkanethiol SAMs were carried out with the nanoparticle on mirror (NPoM) geometry. Figure S5 shows the SERS spectra from this ODT SAM on a particular NPoM, taken over time. This shows the much greater spectral stability of the ODT SAM compared to the lipid system. Results are similar at different times and for different NPoMs.

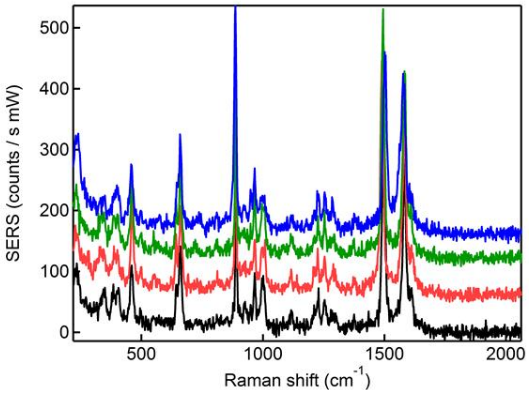

Figure S5 | Nanoparticle on mirror measurements of ODT. SERS spectra of an ODT alkanethiol SAM in the nanoparticle on mirror geometry taken at consecutive times. Laser 633nm, 1mW, 1s integration time.

S5. Additional SERS Tracking data

Figure S6 shows additional fast SERS scans showing fast dynamics of the lipid molecules. These all evidence the same sorts of features discussed in the main text, with changes in intensity, and correlated and anti-correlated spectral shifts. These demonstrate the typical results seen for every NPoM in this geometry with the red scattering spectrum, and show the robustness of our approach to track single molecule dynamics in SERS. The hybrid lipid bilayer is in every case the POPC:DOTAP 80:20 construct shown above.

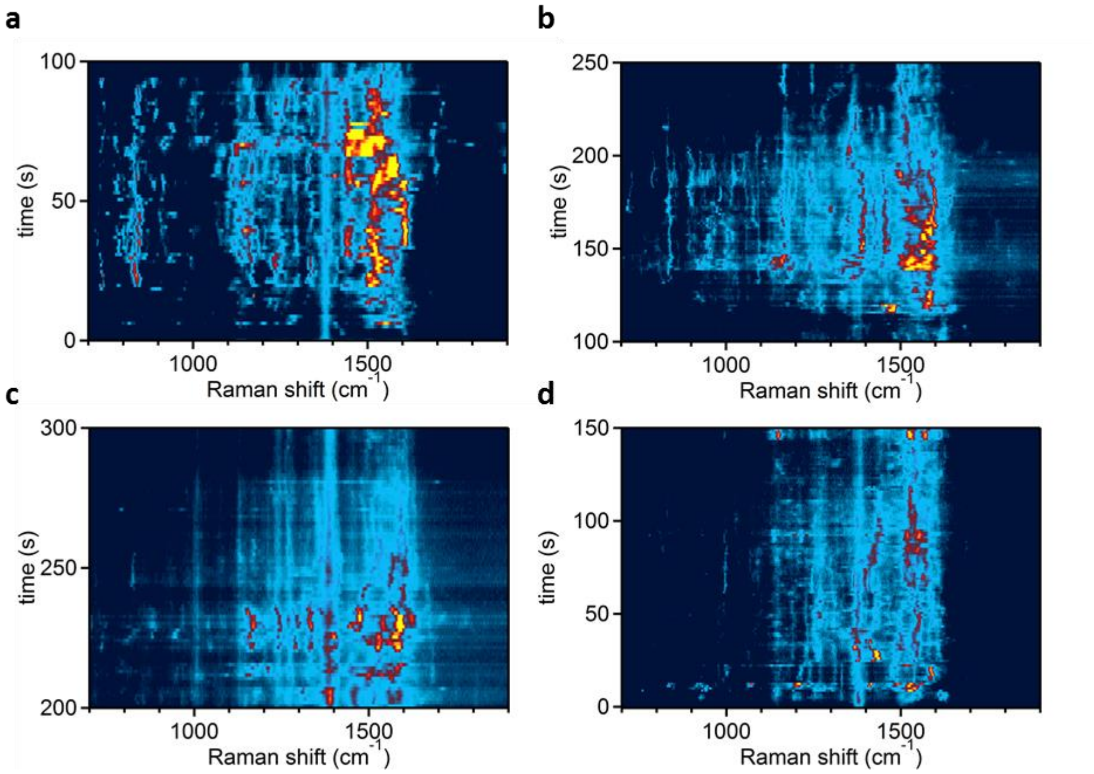

Figure S6 | Fast dynamics of hybrid lipid bilayer SERS. a-d, Time resolved SERS spectra from different gold nanoparticles, showing correlated and anti-correlated mode-shift dynamics. Laser 633nm, 1mW, 1s integration time.

S6. Mode Histograms

Distributions of all Raman vibration mode positions were calculated over a large data set (1200 spectra, on different nanoparticles). Figure S7 shows the histograms for the three correlated lines analysed in Figure 3(d) in the main text.

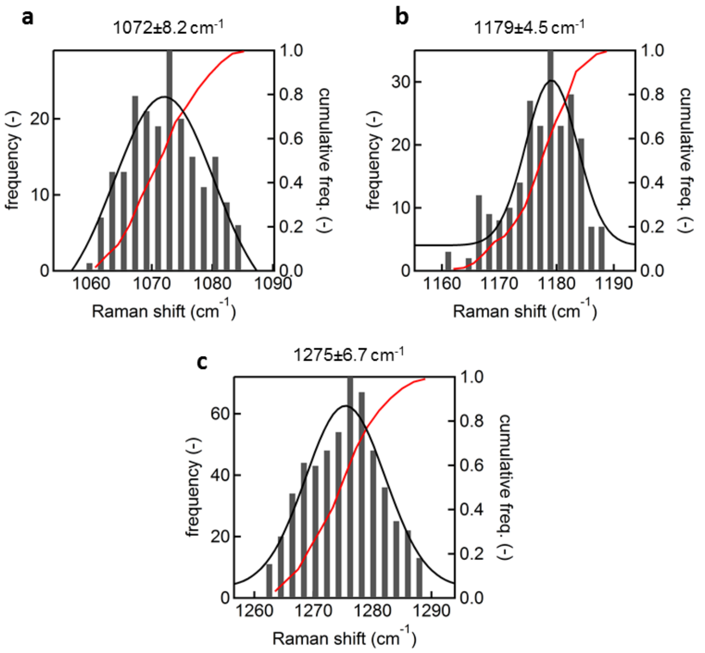

Figure S7 | Mode position distribution curves. a-c, Distribution of Raman line spectral positions for lines at 1072, 1179 and 1275 cm^{-1} over 1200 SERS scans.

S7. Gap plasmon simulation

Electromagnetic field intensities in the NPoM geometry were modelled with the boundary element method using BEMAX. Figure S8 shows the light intensity $|E|^2$ within the gap in lateral and vertical cross-sections at 633nm, corresponding to the Raman excitation wavelength. The obtained field enhancement is $|E|^2 \sim 10^3$, which leads to a realistic Raman scattering enhancement of $|E|^4 \sim 10^6$ for molecules in this spatial location. We can estimate that the SERS signal is produced only from molecules within the full-width half-maximum (FWHM) ~8nm (Fig.S8c) central part of the enhanced region. With a lipid molecule typically occupying an area of 5Å x 5Å, we estimate ~150 molecules in the bilayer might be able to contribute to the SERS. For the added PTPC in 5% concentration, the number of molecules in the hot-spot is hence < 5-10 molecules at any time.

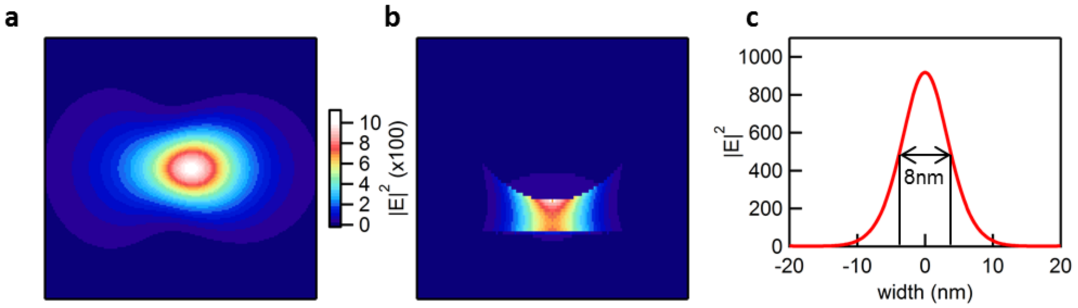

Figure S8 | **Electromagnetic field intensity simulations. a,** Lateral cross-section and **b,** vertical cross-section of simulated field intensity $|E|^2$ in the nanogap. **c,** Field enhancement $|E|^2$ in lateral direction. The FWHM of the 'hot-spot' is ~8nm.

S8. DFT simulations

DFT simulations of a POPC molecule are performed in order to support the mode assignment. The head and tail of the POPC molecule are separately pre-optimised by MOPAC2012[S1] using the PM7[S2,S3] semi-empirical Hamiltonian. The main optimisation is carried out using the Amsterdam Density Functional Theory package[S4-S6] with the triple Z + 1 polarisation function basis set. A frozen core situation is applied to all DFT simulations. Raman active modes are chosen by numerical differentiation of the polarisation tensor.[S7-S9] The extracted peaks were used to help match with literature SERS and the experimental data.

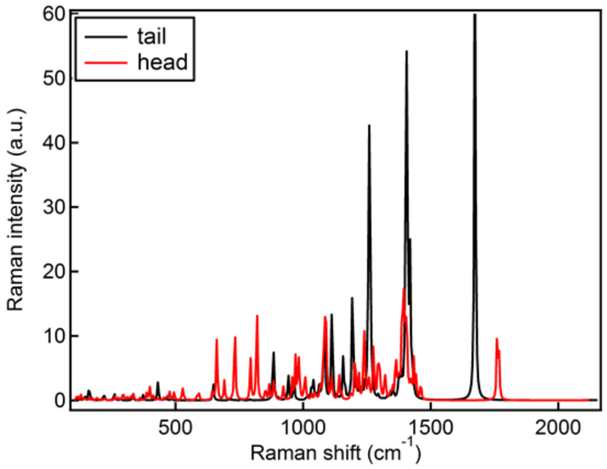

Figure S9 | Simulated Raman spectra of hydrocarbon tails and head group of the phospholipid POPC. Raman spectra calculated with the Amsterdam Density Functional Theory package for the head and tail of a POPC molecule.

S9. Formation of the NPoM geometry

Besides the clear evidence from spectroscopy that these Au NPoM are mostly formed by well separated Au NPs, additional support comes from scanning electron microscopy (SEM). The SEM images below show well separated gold 80nm nanoparticles on an alkanethiol molecular spacer layer produced under equivalent conditions to the HBL system. It is problematic to use an HBL spacer layer for such measurements as the lipids are stripped off the alkanethiol surface when the solvent is lost.

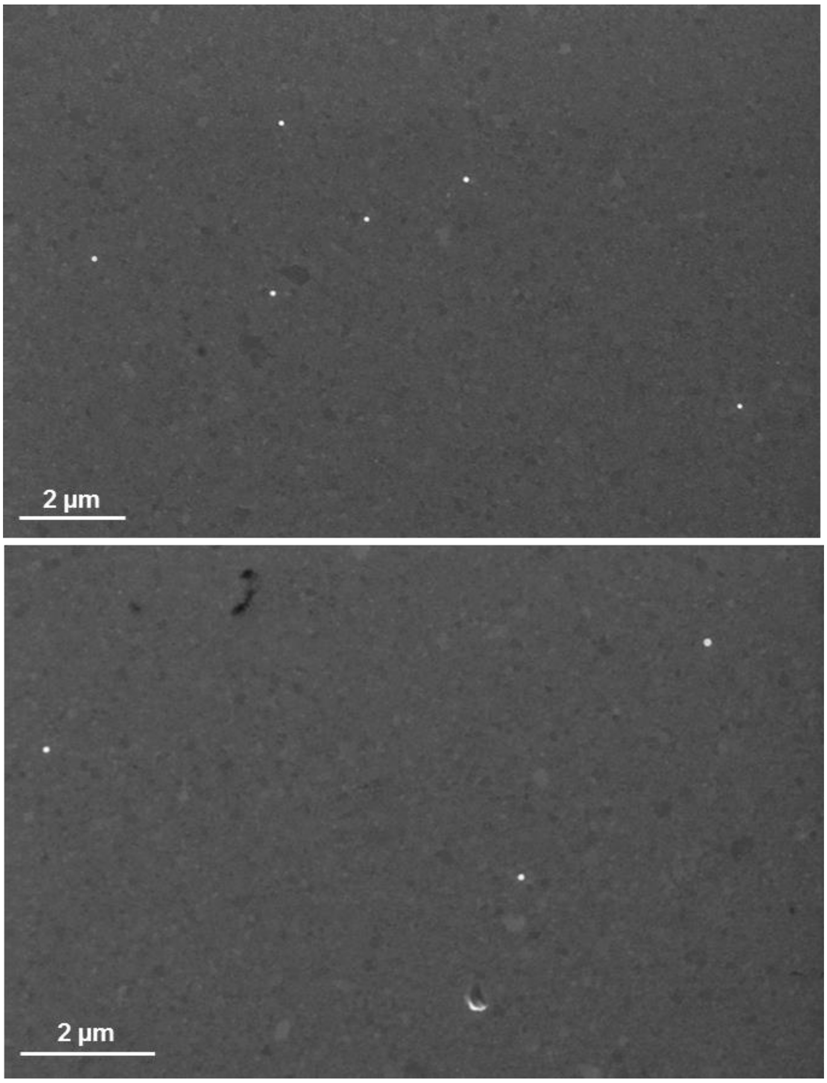

Figure S10 | Scanning Electron Microscopy of gold nanoparticles on an alkanethiol SAM. Images were recorded with an acceleration voltage of 15 kV and a working distance of 2.2 mm.

References

S1. MOPAC2012, Stewart, J. J. P., Stewart Computational Chemistry, Colorado Springs, CO, USA, http://OpenMOPAC.net (2012).

S2. Stewart, J. J. P., Optimization of Parameters for Semiempirical Methods V: Modification of NDDO Approximations and Application to 70 Elements. *J. Mol. Modeling* **13**, 1173-1213 (2007).

S3. Stewart, J. J. P., Optimization of parameters for semiempirical methods VI: more modifications to the NDDO approximations and re-optimization of parameters. *J. Mol. Modeling* **19**, 1-32 (2013).

S4. te Velde, G. *et al.*, Chemistry with ADF. *J. Comput. Chem.***22**, 931 (2001)

S5. Fonseca Guerra, C., Snijders, J.G., te Velde, G. & Baerends, E.J., Towards an order-N DFT method. *Theor. Chem. Acc.* **99**, 391 (1998).

S6. ADF2013, SCM, Theoretical Chemistry, Vrije Universiteit, Amsterdam, The Netherlands, http://www.scm.com.

S7. Fan, L. & Ziegler, T., Application of density functional theory to infrared absorption intensity calculations on main group molecules. *J. Chem. Phys.* **96**, 9005 (1992).

S8. van Gisbergen, S.J.A., Snijders, J.G. & Baerends, E.J., Application of time-dependent density functional response theory to Raman scattering. *Chem. Phys. Lett.* **259**, 599 (1996).

S9. van Gisbergen, S.J.A., Snijders, J.G. & Baerends, E.J., Implementation of time-dependent density functional response equations. *Comput. Phys. Commun.* **118**, 119 (1999).
